# Location of Zeros of Wiener and Distance Polynomials

**DOI:** 10.1371/journal.pone.0028328

**Published:** 2012-03-16

**Authors:** Matthias Dehmer, Aleksandar Ilić

**Affiliations:** 1 Institute for Bioinformatics and Translational Research, UMIT – The Health and Lifesciences University Hall/Tyrol, Hall in Tyrol, Austria; 2 Faculty of Sciences and Mathematics, University of Niš, Niš, Serbia; Philipps-University Marburg, Germany

## Abstract

The *geometry of polynomials* explores geometrical relationships between the zeros and the coefficients of a polynomial. A classical problem in this theory is to locate the zeros of a given polynomial by determining disks in the complex plane in which all its zeros are situated. In this paper, we infer bounds for general polynomials and apply classical and new results to graph polynomials namely Wiener and distance polynomials whose zeros have not been yet investigated. Also, we examine the quality of such bounds by considering four graph classes and interpret the results.

## Introduction

Numerous graph polynomials have been extensively studied and applied interdisciplinarily, see, e.g., [1]–[4]. Early contributions in this area deal with studying the well known independence polynomial [5] and chromatic polynomial [6]. Other graph polynomials such as the Omega polynomial and Cluj polynomial have been studied in [7]. Apart from this research, polynomials have been also employed in biologically driven disciplines. For instance, Emmert-Streib [8] tackled the challenging problem of calculating knot polynomials of secondary structure elements of proteins algorithmically. Related work can be also found in [8]. Interestingly, the development of so-called topological indices such as the well-known Wiener index [9] has triggered exploring graph polynomials too. For instance, Yan et al. [10] examined how the Wiener index changes under certain graph operations and extended their results to Wiener polynomials. Zadeh et al. [11] also investigated Wiener-type invariants of some graph operations. But note that the first paper exploring the change of the Wiener number upon operations on graphs has been contributed by Polansky and Bonchev [12]. Further, formulas for the Wiener polynomial of 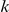-th power graphs have been investigated [13] when considering special graph classes such as paths, cycles and hypercubes (see also Theorem (1)).

In general, graph polynomials have been developed for measuring combinatorial graph invariants and for characterizing graphs. The latter problem has been studied in structural chemistry where the polynomials have been derived from chemical graphs [1], [3]. There, graphs have been characterized by several graph polynomials [14] to solve problems in the Hückel-molecular orbital theory and in the theory of aromaticity, see [3], [15]. Another intriguing field deals with investigating graph measures derived from the zeros of a graph polynomial. Seminal work has been done by Lovász et al. [16] as they explored the meaning of the largest eigenvalue of trees. Particularly they found that the leading positive eigenvalue of the characteristic polynomial can be used as a measure for detecting branching of trees. Related concepts of branching based on using the eigenvalues of a graph have been studied by Randić et al. [17] and Bonchev [18].

Later, Randić et al. [17] surveyed further eigenvalue-based measures such as the sum of the positive eigenvalues, the multiplicity of the zero eigenvalue and other spectral indices [17], [19]. Also, Dehmer et al. [20] recently developed novel spectral measures that turned to be unique for several graph classes. Altogether this shows that graph polynomials and their zeros have been a valuable source for investigating various problems in discrete mathematics and related areas.

Apart from the research described above, the zeros of some graph polynomials have been also explored, see, e.g., [21]–[23]. In this sense, Woodall [23] explored the zeros and zero-free regions of chromatic and flow polynomials. Also, the zero distribution of chromatic and flow polynomials of graphs and characteristic polynomials of matroids have been examined by Jackson [21]. Finally Brešar et al. [24] examined the zeros of cube polynomials under certain structural conditions of the underlying graphs. Other results about the zeros of known graph polynomials have been recently reported by Ellis-Monaghan et al. [4].

The main contribution of this paper is twofold: First, we prove inclusion radii representing upper bounds for the zeros of general complex polynomials. Note that most of these statements can also be applied if the polynomials possess real coefficients as the moduli of the coefficients appear in the corresponding bounds. Second, we apply these and classical results to locate the zeros of special Wiener and distance polynomials, see [25]–[27]. This results in disks in the complex plane or intervals where the zeros of these polynomials lie. To our best knowledge, the location of zeros of the Wiener and distance polynomial has not been studied yet. Apart from proving results for special polynomials, i.e., the polynomials represent special graph classes, it is easy to generalize the results for other (general) graph polynomials by using the tools we will provide in this paper. Besides further developing the mathematical apparatus, we evaluate the quality of the zero bounds by generating four large graph classes and interpret the numerical results.

## Results

The main contribution of this paper is to locate the zeros of special graph polynomials which have been proven useful in mathematical chemistry and discrete mathematics, see [3], [14], [25]. A thorough overview of the underlying theory called *analytic theory of polynomials* can be found in [28], [29]. Note that the problem of finding bounds for the zeros of complex and real polynomials has been tackled by numerous authors, e.g., see [28], [30]–[34]. However, the existing research shows that the usefulness and performance of many such bounds has not been demonstrated yet. For this, we compare our bounds in the section ‘Numerical Results’ and demonstrate that some of the new bounds are optimal.

We now start by reproducing some important definitions and results we are going to use in our analysis.

### Mathematical Preliminaries

In this section, we introduce some mathematical preliminaries [25]–[27], [35], [36]. Let 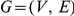 be a finite simple graph and let 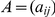 be its adjacency matrix. 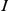 denotes the identity matrix. Then,
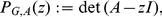
(1)is the characteristic polynomial of 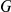. Straightforwardly, we obtain the distance polynomial defined by
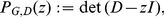
(2)where 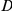 is the distance matrix of 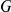. By expanding the determinant, we yield
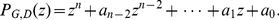
(3)We see that 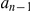 is always equal to zero [26]. Denote by 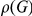 the diameter of 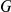 and 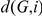 is the number of pairs of 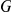 having distance 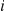, 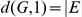. Then the Wiener polynomial [25], [27] (also called Hosoya polynomial [37]) can be defined as
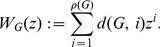
(4)Further properties of 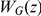 have been reported in [27]. Next, we reproduce some results due to Sagan et al. [27] and K
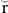
ivka [26] giving concrete expressions for Wiener- and distance polynomials for special graph classes.

**Theorem 1** *Let* 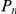, 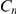 *and* 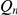 *be the path graph, cycle graph and* 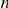 *-dimensional cube. It holds*

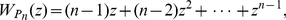
(5)

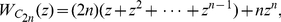
(6)

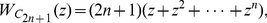
(7)

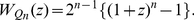
(8)


**Theorem 2** *Let* 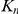 *and* 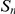 *be the complete graph and the star graph on* 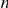 *vertices. It holds*

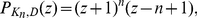
(9)

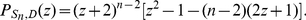
(10)


To introduce the problem of locating the zeros of polynomials, we state the following definitions.

**Definition 1** *Let*

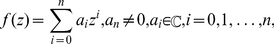
(11)
*be complex polynomial. The set*

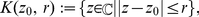
(12)
*represents a circle with central point* 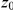 *and radius* 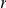. *Further, we define*

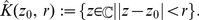
(13)


**Definition 2** *If all zeros of* 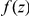 *lie in the set given by* *Equation (12)* *,* 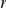 *is called the inclusion radius. In the simplest case,* 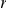 *is a function of all coefficients, i.e.,* 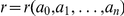.

Note that a more general question namely deriving bounds depending on 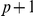 coefficients for 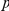 zeros of 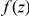 has been tackled by Montel [28], [38]. Other variants of bounds and extensions of the results due to Montel can be also found in [28].

### Known Inclusion Radii

In this section, we state some classical and known results for locating the zeros of arbitrary complex-valued polynomials.

**Theorem 3 (Cauchy** [**28**] **)** *Let*

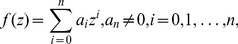
(14)
*be complex polynomial. All zeros of* 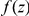 *lie in* 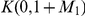, *where*

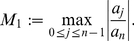
(15)


**Theorem 4 (Fujiwara** [**39**] **)** *Let*

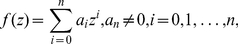
(16)
*be complex polynomial. For* 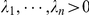 *and* 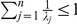, *all zeros of* 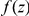 *lie in*

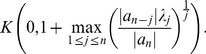
(17)


**Theorem 5 (Enestrom-Kakeya** [**40**] **)** *Let*

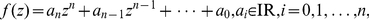
(18)
*be a polynomial with real coefficients satisfying*

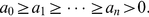
(19)
*Then, no zeros of f(z) lie in* 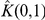.

**Theorem 6 (Dehmer** [**41**] **)** *Let*

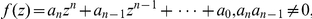

*be a complex polynomial. All zeros of* 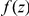 *lie in the closed disk*

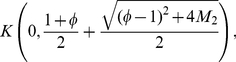
(20)where
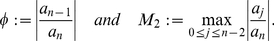
(21)


Besides locating the zeros of polynomials, it is often important to determine the number of positive or negative zeros of polynomials with real coefficients. In this light, we state the famous Descartes Rule of Signs, see [28], [34].

**Theorem 7** *Let f(z) be a real polynomial. The number of positive zeros of* 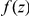 *either equals the number of sign changes within the sequence of coefficients or is less than it by a multiple of two.*

### Novel Inclusion Radii

**Theorem 8** *Let*

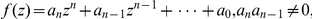

*be a complex polynomial. All zeros of* 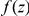 *lie in the closed disk*

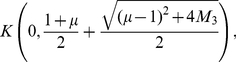
(22)where
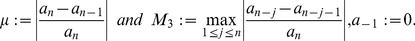
(23)


**Proof**: Defining 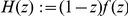 and assuming 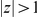 yields
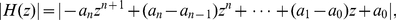
(24)

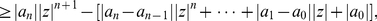
(25)

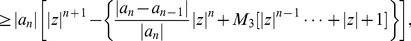
(26)

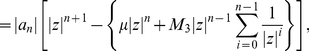
(27)

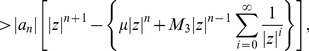
(28)

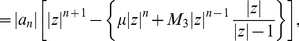
(29)

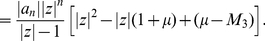
(30)We set
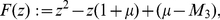
(31)and conclude 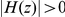 if 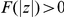. To solve 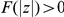, we yield
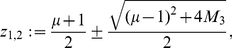
(32)and see that
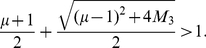
(33)Altogether, we obtain
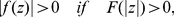
(34)and, finally
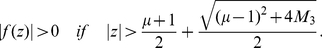
(35)By using Inequality (33), it is evident that the zeros with 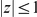 lie in the closed disk represented by Equation (22) too. The theorem is proven for 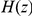. But all zeros of 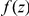 are zeros of 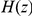. Hence, the theorem also holds for 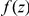. □

**Theorem 9** *Let*

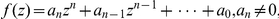
be a complex polynomial. Define
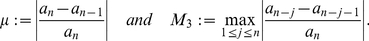
(36)All zeros of 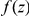 lie in the closed disk 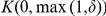 where 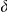 denotes the positive root of the equation
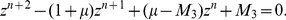
(37)


**Proof**: Defining 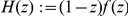 yields again
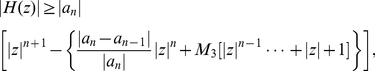
(38)

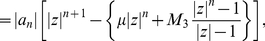
(39)

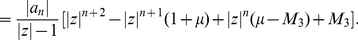
(40)We set
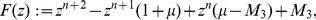
(41)and see 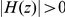 if 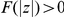. In both cases, i.e., 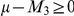 and 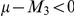, 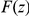 has two sign changes in its sequence of coefficients. By applying Theorem (7) and observing 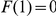 and 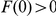, we conclude that 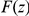 has exactly two positive zeros. Let 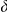 be the zero
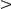
1 and 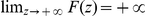. Altogether, we obtain
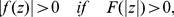
(42)and, finally
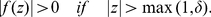
(43)The proof for 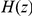 is complete. But all zeros of 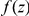 are zeros of 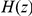. Hence, the theorem also holds for 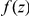. □

The next theorem is based on using the Hölder inequality [42].

**Theorem 10** *Let*

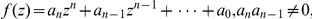

*be a complex polynomial. Let* 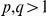 *such that* 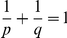 *and define*

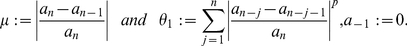
(44)
*All zeros of* 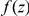 *lie in the closed disk* 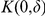 *where* 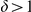 *denotes the largest positive root of the equation*

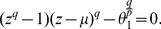
(45)


**Proof**: We start with 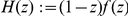 and obtain
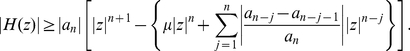
(46)By applying the well-known Hölder inequality [42] to 
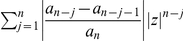
 and 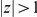, we further infer
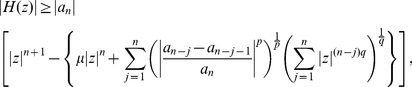
(47)

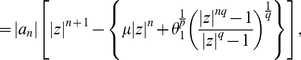
(48)

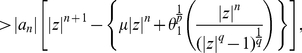
(49)

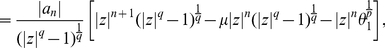
(50)

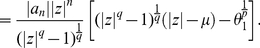
(51)Hence, 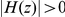 if
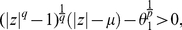
(52)or
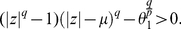
(53)Define
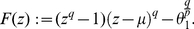
(54)We see easily that the largest positive zero 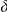 of 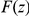 is 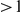. This implies 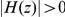 if 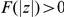 and, hence, 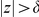. Thus, we proved the theorem for 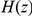. But all zeros of 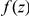 are zeros of 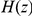. □

**Corollary 1** *Let*

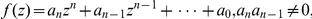

*be a complex polynomial.* 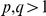 *and* 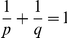. *If* 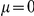 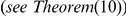, *all zeros of* 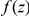 *lie in the closed disk* 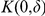 *where* 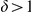 *denotes the largest positive root of the equation*

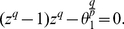
(55)


**Proof**: Set 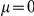 in Equation (54). □

The following theorem holds for polynomials with real coefficients and was proven to be optimal by using several graph classes (see section ‘Numerical Results’).

**Theorem 11** *Let*

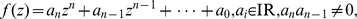

*be a polynomial with real coefficients. Define*

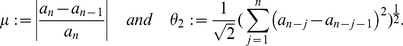
(56)
*All zeros of* 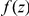 *lie in the closed disk* 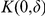 *where* 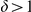 *denotes the largest positive root of the equation*

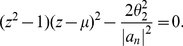
(57)


**Proof**: Define 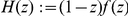. We obtain
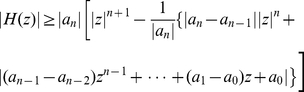
(58)

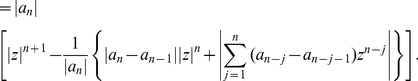
(59)Now, we use De Bruijn's inequality [42] given by
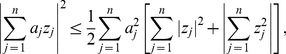
(60)where 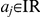 and 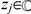. Applying this inequality to 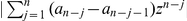 yields
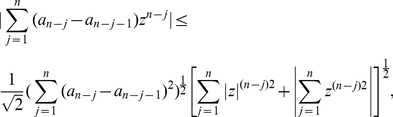
(61)

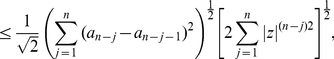
(62)

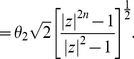
(63)By using the last inequality and assuming 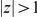, we further obtain
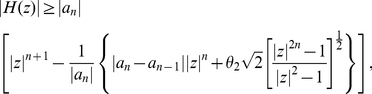
(64)

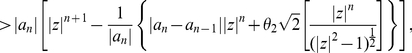
(65)

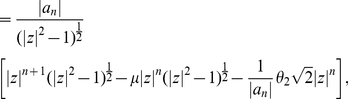
(66)

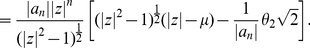
(67)Thus, 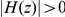 if
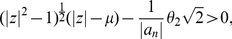
(68)or
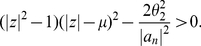
(69)Again, we define
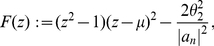
(70)and easily observe that its largest positive zero 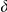 is 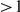. Finally, 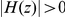 if 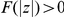 and, hence, 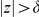. Thus, we completed the proof for 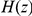. As the zeros of 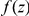 are zeros of 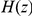, the proof of the theorem is complete. □

Now, we easily obtain the following corollaries.

**Corollary 2** *Let*

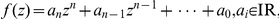

*be a polynomial with real coefficients. All zeros of* 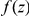 *lie in the closed disk* 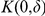 *where* 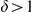 *denotes the largest positive root of the equation*

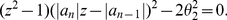
(71)


**Proof**: The statement follows from applying the steps of the proof of Theorem (11) to 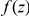 (instead of starting with 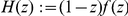. □

**Corollary 3** *Let*

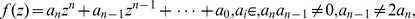

*be a polynomial with real coefficients. All zeros of* 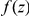 *lie in the closed disk*

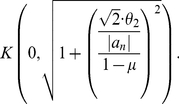
(72)


**Proof**: Using Inequality (67) and 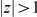 yields
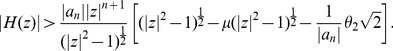
(73)Now, 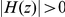 if
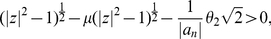
(74)or
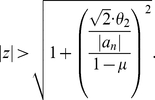
(75)It holds 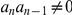. Then, 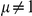 iff 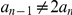. □

**Corollary 4** *Let*

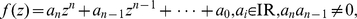

*be a polynomial with real coefficients. If* 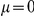, *all zeros of* 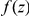 *lie in the closed disk*

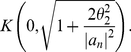
(76)


**Proof**: Set 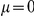 in Inequality (73). The rest of the proof is analogous to the proof of Corollary (3). □

### Location of Zeros of Graph Polynomials

By using the tools presented in the previous section, we are now able to derive results for locating the zeros of Wiener and distance polynomials.

### Bounds for Concrete Graph Polynomials

We start by considering the polynomials provided in section ‘Mathematical Preliminaries and Known Results’ (see Theorem (1)).

**Corollary 5** 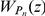, 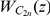, 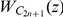 *and* 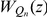 *do not possess positive zeros.*

**Proof**: As there are no sign changes in the sequences of the coefficients of 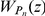, 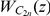 and 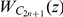, the assertion follows immediately by applying Theorem (7). To prove the statement for 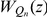, we easily see that
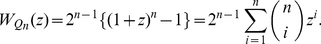
(77)Again by applying Theorem (7), 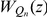 does not possess positive zeros. □

**Remark 12** *The number of negative zeros of these graph polynomials can be determined by applying Theorem (7) to* 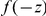. *Particularly,* 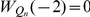 *if* 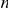 *is even.*

Next, we apply the Theorem of Eneström-Kakeya [40] and obtain the following corollary.

**Corollary 6** 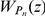 *and* 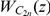 *do not possess zeros in* 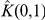.

To derive a more detailed statement for the zeros of 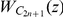, we firstly state a lemma.

**Lemma 1** *Let*

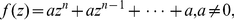
(78)
*be a complex polynomial. All zeros of* 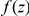 *lie on the unit circle.*

**Proof**: Clearly, we have
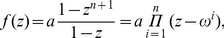
(79)where 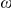 denotes the 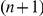-th root of unity. The lemma is proven. □

**Corollary 7** *All zeros of* 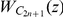 *lie on the unit circle.*

By applying the classical result due to Cauchy (see Theorem (3)), we obtain

**Corollary 8** *All zeros of* 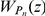, 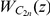 *and* 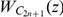 *lie in* 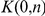, 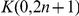 *and* 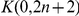, *respectively.*

By applying Theorem (6), we also yield

**Corollary 9** *All zeros of* 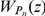, 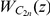 *and* 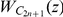 *lie in*

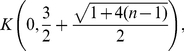
(80)

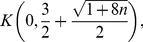
(81)
*and*

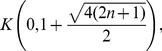
(82)
*respectively.*

For 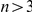, we yield
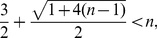
(83)since it is equivalent to
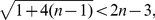
(84)and
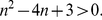
(85)This inequality is satisfied for 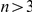 and, hence, the inclusion radius given by Equation (80) is always an improvement of 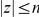 (see Corollary (8)). This relation can be proven analogously for the other zero bounds too (see Equation (81), (82) and Corollary (8)).

As for 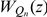 no special conditions for its coefficients hold, Eneström-Kakeya's Theorem is not applicable. Theorem (3) and Theorem (6) give general zero bounds for 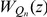.

**Corollary 10** *All zeros of* 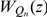 *lie in*

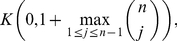
(86)
*and*

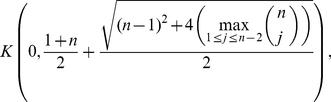
(87)
*respectively.*

We notice that the maximum of 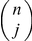 is achieved for the middle binomial coefficient 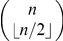.

Theorem (11) turned out to be feasible for various graph classes (see section ‘Numerical Results’). Hence, we apply this statement to some of the Wiener polynomials of Theorem (1). Note that the bound given by Theorem (11) represents a so-called implicit bound as the bound value is a root of a concomitant polynomial, see, e.g., Equation (57).

**Corollary 11** *All zeros of* 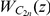 *lie in the closed disk* 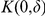 *where* 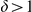 *denotes the largest positive root of the equation*

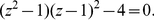
(88)It is 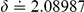.

**Corollary 12** *All zeros of* 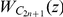 *lie in the closed disk* 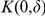 *where* 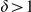 *denotes the largest positive root of the equation*

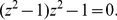
(89)It is 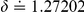.

**Corollary 13** *All zeros of* 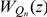 *lie in the closed disk* 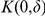 *where* 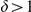 *denotes the largest positive root of the equation*

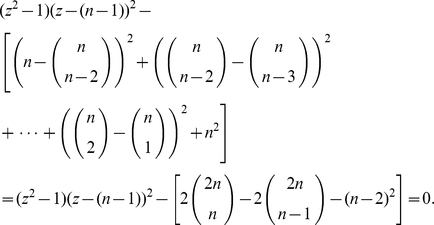



Before applying the results from the previous section to the special distance polynomials presented in Theorem (2), we state a simple lemma.

**Lemma 2**

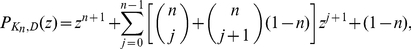
(90)

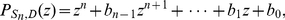
(91)
*where*

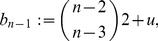
(92)

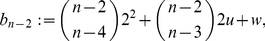
(93)

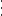


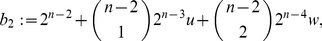
(94)

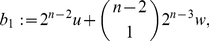
(95)

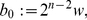
(96)

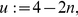
(97)

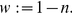
(98)


**Proof**: We start with 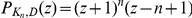, see Theorem (1). By performing direct calculations, we get
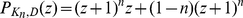
(99)

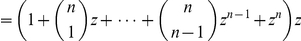


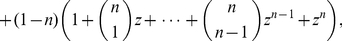
(100)

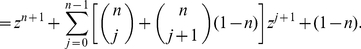
(101)Now, consider 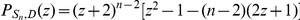. In order to infer Equation (91), we observe
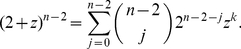
(102)Also,
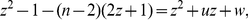
(103)where 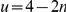 and 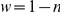. If we now define
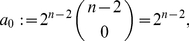
(104)

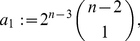
(105)

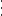


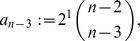
(106)

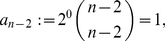
(107)we yield
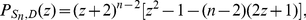
(108)

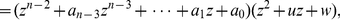
(109)

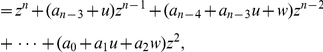


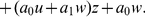
(110)With the definitions stated in Lemma (2) and 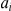, 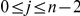 expressed above, we obtain
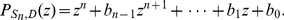
(111)□

To finalize this section, we now apply some of the classical and new results to the special distance polynomials stated in Lemma (2). Note that these polynomials only possess real zeros as the underlying matrices are symmetric (see Definition (2)). We state the results exemplarily by only considering 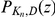.

Using Theorem (3) yields

**Corollary 14** *All zeros of* 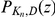 *lie in the interval* 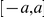, *where*

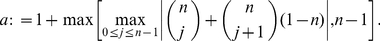
(112)


Applying Theorem (6) yields

**Corollary 15** *All zeros of* 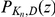 *lie in the interval* 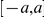, *where*

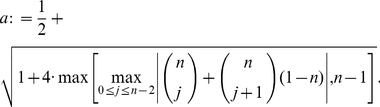
(113)


Finally we apply Theorem (11) and obtain

**Corollary 16** *All zeros of* 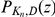 *lie in the interval* 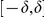 *where* 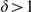 *denotes the largest positive root of the equation*

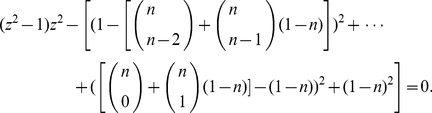
(114)


It is evident that by using Lemma (2), similar statements can be derived for 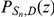.

### Numerical Results

In this section, we evaluate the quality of the zero bounds presented in the previous sections. Note that this problem is challenging when no sharpness results are available. That means given several bounds and classes of polynomials, we have to judge what kinds of bounds are best for a particular class. To solve this problem analytically might be feasible for bounds which are based on the same concept, e.g., zero bounds as functions of all coefficients which can be calculated explicitly (explicit bounds). But if we consider bounds defined on different concepts, a comparison is often difficult without determining the bounds for concrete polynomials.

To tackle this problem for some zero bounds presented in this paper, we use special graph classes whose graph polynomials and their real and complex-valued zeros can be directly calculated. To generate these graph classes, we have used the well-known Nauty package, see [43]. The package Nauty is a program for computing automorphism groups of graphs and digraphs, written in a highly portable subset of the language C. This package also includes a suite of programs called gtools for efficiently generating and processing small non-isomorphic graphs (stored in graph6 format) with various constrains, such as the number of vertices, edges, maximum/minimum vertex degree, connectedness, etc. Now we define the graph classes as follows:

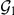
: Unicyclic graphs with 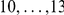 vertices. 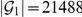.

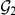
: Connected graphs with 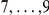 vertices. 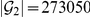.

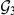
: Bicyclic graphs. with 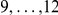 vertices. 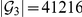.

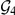
: Trees with 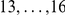 vertices. 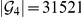.

**Figure 1:**
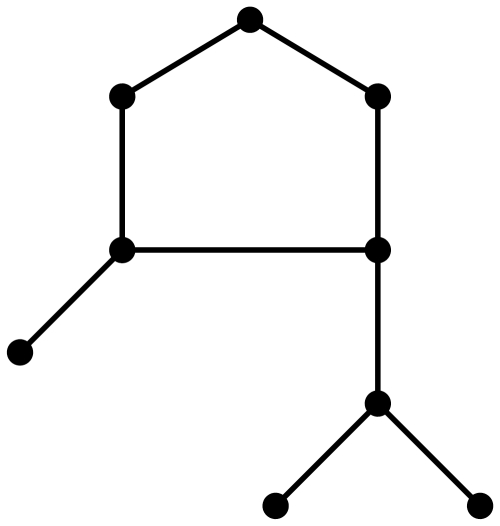
A graph 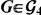.

Note that a tree is a connected graph without cycles, or a connected graph with exactly 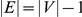 edges. A unicyclic graph is a connected graph with exactly one cycle, or a connected graph with exactly 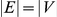 edges. Analogously, a bicyclic graph is a connected graph with exactly two cycles, or a connected graph with exactly 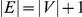 edges. These simple types of graphs have often been used in mathematical chemistry and as underlying structure of chemical compounds. From these characterizations, the most important structural properties of our graph classes are known. Some characteristic graphs from the graph classes 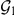, 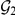, 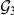 and 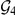 are depicted in Figure (1)–[Fig pone-0028328-g002] [Fig pone-0028328-g003] (4).

**Figure 2 pone-0028328-g002:**
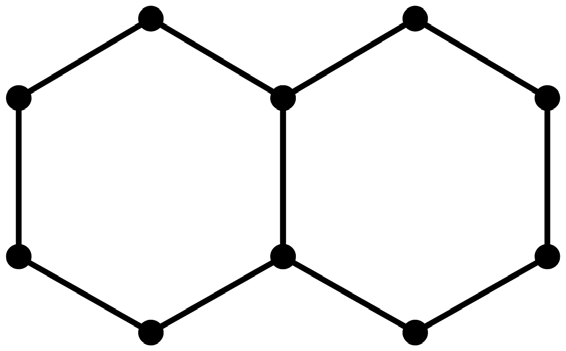
A graph 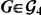.

**Figure 3 pone-0028328-g003:**
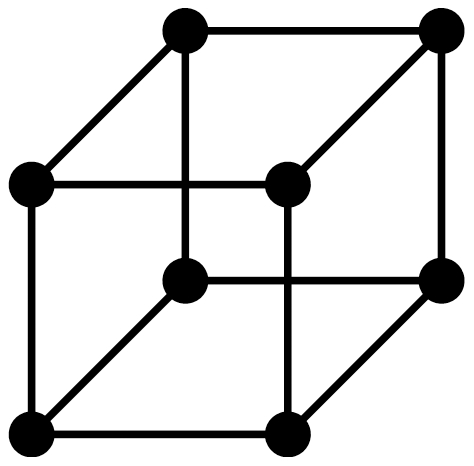
A graph 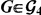.

**Figure 4:**
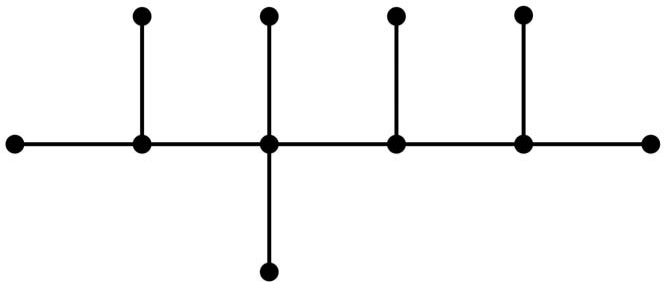
A graph 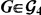.

The numerical results are presented in Table (1)–[Table pone-0028328-t002] [Table pone-0028328-t003] Table (4). 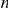 denotes the number of vertices. The mean and standard deviation have been calculated based on the values for the particular graph class. ‘Count best’ stands for the number of graphs for which the particular bound is the best one among all considered bounds. Among the bounds presented in this paper, we also calculated the bound due to Fujiwara [39], see Theorem (4). The first line in each group is ‘Maximum root’, which stands for the statistics regarding the maximum root of distance polynomial computed with 10 digit precision. Note that these values are used for the comparison with other bounds and ‘Count best’ is exactly the number of graphs in the group. Because of ties, the sum of the numbers in the column ‘Count best’ does not need to match up with ‘Count best’ for ‘Maximum root’ row.

**Table 1:** Comparison of the bounds for 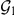.

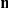	Bound	Mean	St. Deviation	Count best
10	Maximum root	2.007014	0.0	657
	Cauchy (Theorem (3))	7.408803	6.486541	0
	Theorem (6)	5.110194	3.261665	0
	Theorem (8)	4.220755	2.376921	0
	Theorem (9)	3.709663	1.915489	108
	Theorem (11)	3.592285	1.845886	547
	Fujiwara (Theorem (4))	6.012736	4.584253	2
11	Maximum root	2.028913	0.0	1806
	Cauchy (Theorem (3))	8.236638	7.515486	0
	Theorem (6)	5.300828	3.440242	0
	Theorem (8)	4.343894	2.487811	0
	Theorem (9)	3.835794	2.027818	468
	Theorem (11)	3.749178	1.992102	1315
	Fujiwara (Theorem (4))	6.170153	4.732012	23
12	Maximum root	2.063067	0.0	5026
	Cauchy (Theorem (3))	9.129134	8.615679	0
	Theorem (6)	5.494651	3.611637	0
	Theorem (8)	4.473329	2.594855	0
	Theorem (9)	3.967472	2.136432	1514
	Theorem (11)	3.910585	2.131267	3446
	Fujiwara (Theorem (4))	6.333819	4.874544	62
13	Maximum root	2.103169	0.0	13999
	Cauchy (Theorem (3))	10.06646	9.773008	0
	Theorem (6)	5.684568	3.772948	0
	Theorem (8)	4.599854	2.689574	0
	Theorem (9)	4.095588	2.231771	5087
	Theorem (11)	4.068939	2.259367	8684
	Fujiwara (Theorem (4))	6.488192	5.005889	220

**Table 2 pone-0028328-t002:** Comparison of the bounds for 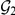.

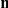	Bound	Mean	St. Deviation	Count best
7	Maximum root	2.751998	0.0	853
	Cauchy (Theorem (3))	5.471599	3.603993	0
	Theorem (6)	4.665022	2.360952	1
	Theorem (8)	4.461638	1.919881	1
	Theorem (9)	3.870582	1.459967	11
	Theorem (11)	3.71606	1.351209	839
	Fujiwara (Theorem (4))	6.887799	5.225005	4
8	Maximum root	3.641017	0.0	11117
	Cauchy (Theorem (3))	6.74994	4.194339	0
	Theorem (6)	5.762259	2.535831	1
	Theorem (8)	5.460469	2.016927	1
	Theorem (9)	4.876477	1.553454	298
	Theorem (11)	4.74001	1.457854	10811
	Fujiwara (Theorem (4))	8.955896	6.841888	9
9	Maximum root	4.970174	0.0	261080
	Cauchy (Theorem (3))	8.22829	4.572521	0
	Theorem (6)	7.135768	2.561838	1
	Theorem (8)	6.780506	1.999698	1
	Theorem (9)	6.198375	1.535316	12046
	Theorem (11)	6.083267	1.460323	248967
	Fujiwara (Theorem (4))	11.705689	8.867751	68

**Table 3 pone-0028328-t003:** Comparison of the bounds for 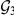.

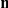	Bound	Mean	St. Deviation	Count best
9	Maximum root	2.22706	0.0	797
	Cauchy (Theorem (3))	7.298504	6.051353	0
	Theorem (6)	5.279362	3.200967	0
	Theorem (8)	4.4397	2.362881	0
	Theorem (9)	3.924488	1.895787	134
	Theorem (11)	3.796987	1.811045	658
	Fujiwara (Theorem (4))	6.537748	4.913553	5
10	Maximum root	2.180459	0.0	2678
	Cauchy (Theorem (3))	8.143751	7.218366	0
	Theorem (6)	5.447246	3.437328	0
	Theorem (8)	4.525308	2.522573	0
	Theorem (9)	4.014815	2.061737	609
	Theorem (11)	3.918314	2.016088	2045
	Fujiwara (Theorem (4))	6.60265	5.032024	24
11	Maximum root	2.182083	0.0	8833
	Cauchy (Theorem (3))	9.025023	8.296308	0
	Theorem (6)	5.631895	3.627826	0
	Theorem (8)	4.638066	2.639716	0
	Theorem (9)	4.130523	2.179354	2737
	Theorem (11)	4.069533	2.173708	6029
	Fujiwara (Theorem (4))	6.732582	5.180786	67
12	Maximum root	2.209132	0.0	28908
	Cauchy (Theorem (3))	9.9542	9.412301	0
	Theorem (6)	5.820146	3.793567	0
	Theorem (8)	4.761158	2.739752	0
	Theorem (9)	4.2557	2.279321	10390
	Theorem (11)	4.228459	2.306858	18211
	Fujiwara (Theorem (4))	6.882458	5.316112	302

**Table 4:** Comparison of the bounds for 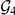.

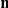	Bound	Mean	St. Deviation	Count best
13	Maximum root	2.013052	0.0	1301
	Cauchy (Theorem (3))	9.120055	8.891774	0
	Theorem (6)	5.368856	3.55083	0
	Theorem (8)	4.326742	2.506002	0
	Theorem (9)	3.821249	2.051564	392
	Theorem (11)	3.773694	2.058365	882
	Fujiwara (Theorem (4))	6.045571	4.656	22
14	Maximum root	2.047998	0.0	3159
	Cauchy (Theorem (3))	10.015765	10.009886	0
	Theorem (6)	5.550602	3.709817	0
	Theorem (8)	4.450377	2.604708	0
	Theorem (9)	3.946445	2.151	1164
	Theorem (11)	3.926453	2.188239	1929
	Fujiwara (Theorem (4))	6.188467	4.773453	59
15	Maximum root	2.077108	0.0	7741
	Cauchy (Theorem (3))	10.922225	11.134163	0
	Theorem (6)	5.722803	3.863504	0
	Theorem (8)	4.566722	2.699576	0
	Theorem (9)	4.063896	2.245945	3166
	Theorem (11)	4.070977	2.312517	4384
	Fujiwara (Theorem (4))	6.317809	4.882851	179
16	Maximum root	2.10139	0.0	19320
	Cauchy (Theorem (3))	11.862751	12.311891	0
	Theorem (6)	5.88555	4.013454	0
	Theorem (8)	4.674308	2.791324	0
	Theorem (9)	4.17225	2.337896	8904
	Theorem (11)	4.206391	2.433625	9917
	Fujiwara (Theorem (4))	6.42628	4.974219	477

It is not surprising that Cauchy's bound (see Theorem (3)) often gives non-feasible values if 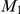 is large. An example for this is the polynomial 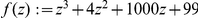, 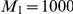. Then Cauchy's bound (see Theorem (3)) gives the closed disk 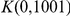. That means the inclusion radius equals 1001 but, in fact, the largest modulus of the zeros of 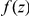 (maximum root) is 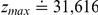. This proves that the resulting bound value is not in accordance with the real location of the zeros of this given polynomial.

Interestingly, the implicit bounds given by Theorem (9) and Theorem (11) clearly outperform the other zero bounds. These statements show even better performance than the bound given by Theorem (6) that has been proven better than other classical results, see [31], [44]. Finally we observe (see Table (1)–[Table pone-0028328-t002] [Table pone-0028328-t003] Table (4)) that Theorem (11) is the best for all graph classes. In particular, we see that the concomitant polynomial of Theorem (11) has degree four. This is a great advantage in practice, since we can use explicit formulas for the largest root of polynomial of degree four and establish sharp upper bounds for the largest root of a distance polynomial.

Generally, we point out that this paper does not deal with calculating the zeros of complex or real polynomials numerically, see [45]. This problem and the task we dealt with in our paper can not compared directly as locating the zeros of a polynomial, e.g., to determine zero bounds does not necessarily require to compute the zeros numerically. For example, Cauchy's bound (see Theorem (3)) and other explicit ones can be determined immediately without using any algorithms, e.g., the method due to Lehmer-Schur to calculate zeros explicitly. Also, many problems do not require to calculate all zeros explicitly as estimations for the zeros are often adequate, e.g., when determining a bound of the largest eigenvalue of a characteristic polynomial, see [16]. But in fact, the analytical methods such as bounds can be useful for using numerical approaches properly as the bound values could be used as starting values.

### Summary and Conclusion

In this paper we have explored the location of zeros of special graph polynomials. Apart from locating the zeros of chromatic and flow polynomials [21], [23], this problem has not yet been investigated extensively for other types of graph polynomials. In this study, we applied classical and new results to locate the zeros of Wiener and distance polynomials representing special graph classes, see [25]–[27]. Clearly, similar statements can be easily obtained for general forms for these polynomials. Also, further theorems can be established by using suitable inequalities from the mathematical literature.

We point out that some of the gained zero bounds might be more practicable than existing results. For example, the root of the concomitant polynomial of Theorem (11) that has degree four can be determined much easier (by hand) than the root of an algebraic equation having degree 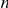. Interestingly, this bound turned out to be optimal for the considered graph classes. Note that zero-free regions for these polynomials could be easily obtained. We will tackle this problem as future work.

The next step was to evaluate the quality of the obtained zero bounds. This is crucial as some practical applications require sharp inclusion radii. Generally, to evaluate the quality of zero bounds relates to determine the bounds by using concrete polynomials or classes thereof. Clearly, it might be difficult to compare explicit and implicit bounds analytically. Thus to derive statements for their optimality, the bounds must be calculated explicitly. In this study, we tackled the problem by using the graph classes 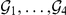 and found that Theorem (9) and Theorem (11) are optimal (see Table (1)–[Table pone-0028328-t002] [Table pone-0028328-t003] Table (4)). As future work, we will perform further studies to explore the optimality of zero bounds. Also, we want to study this problem theoretically and derive optimality statements for certain graph classes.

The meaning of the complex zeros of the Wiener polynomial is not yet understood. To tackle this problem in the future, it would be interesting to use also directed networks for exploring relationships between the complex zeros and certain structural properties of the underlying directed networks, e.g., the *information flow*. Apart from this problem, it would be worthwhile to explore the zero distribution of the Wiener polynomial. A starting point to do so could be employing the seminal work of Schmidt and Schur [34], [46].
